# Association of *BAK1* single nucleotide polymorphism with a risk for dengue hemorrhagic fever

**DOI:** 10.1186/s12881-016-0305-3

**Published:** 2016-07-11

**Authors:** Tran Ngoc Dang, Izumi Naka, Areerat Sa-Ngasang, Surapee Anantapreecha, Nuanjun Wichukchinda, Pathom Sawanpanyalert, Jintana Patarapotikul, Naoyuki Tsuchiya, Jun Ohashi

**Affiliations:** Graduate School of Comprehensive Human Sciences, University of Tsukuba, Tsukuba, Japan; Faculty of Public Health, University of Medicine and Pharmacy at Ho Chi Minh city, Ho Chi Minh City, Viet Nam; Faculty of Medicine, University of Tsukuba, Tsukuba, Japan; Graduate School of Science, The University of Tokyo, Tokyo, Japan; Department of Medical Sciences, Ministry of Public Health, Nonthaburi, Thailand; Food and Drug Administration, Ministry of Public Heath, Nonthaburi, Thailand; Faculty of Tropical Medicine, Mahidol University, Bangkok, Thailand

## Abstract

**Background:**

Dengue hemorrhagic fever (DHF) is a severe life-threatening form of dengue infection. Low platelet count is one of the characteristic clinical manifestations in patients with severe dengue. However, little is known about genetic factors in the host that cause low platelet count in patients with dengue.

**Methods:**

A previous genome-wide association study of hematological and biochemical traits identified single nucleotide polymorphisms (SNPs) associated with low platelet count in healthy subjects. To examine the possible association of these SNPs with DHF, 918 Thai patients with dengue [509 patients with DHF and 409 with dengue fever (DF)] were genotyped for five SNPs: rs5745568 in *BAK1*, rs6141 in *THPO*, rs6065 in *GP1BA*, rs739496 in *SH2B3*, and rs385893 in *RCL1*. In addition, rs4804803 in *CD209*, that has been reported to be associated with dengue infection, was also genotyped to examine if rs4804803 affects the association detected in this study.

**Results:**

The allele frequencies of each SNP were compared between the DHF and DF groups. Among the five SNPs, the G allele of rs5745568 in *BAK1* was significantly associated with a risk for DHF [*P* = 0.006 and crude odd ratio (95 % confidence interval) = 1.32 (1.09–1.60)]. The association of this allele with DHF was also significant in a logistic regression analysis adjusted for age, sex, hospital (i.e., geographic region), immune status (i.e., primary or secondary infection), and virus serotype [*P* = 0.016 and adjusted odd ratio (95 % confidence interval) = 1.29 (1.05–1.58)]. The result was not influenced by rs4804803 [*P* = 0.0167 and adjusted OR (95 % CI) = 1.29 (1.05–1.58)]. No other SNPs including rs4804803 showed significant association.

**Conclusions:**

The low-level constitutive production of platelets caused by the G allele of rs5745568 seems to increase the risk of bleeding in dengue infection. Our results suggest that BCL-2 homologous antagonist/killer (BAK) protein, encoded by *BAK1*, plays a crucial role in the pathogenesis of DHF.

## Background

Dengue is a mosquito-borne acute systemic viral infection caused by the dengue virus (DEN) [1]. DEN is a small single-stranded RNA virus consisting of four distinct serotypes, DEN-1 to DEN-4 [2]. The severity of DEN infection ranges from a mild disease, dengue fever (DF), to the severe dengue hemorrhagic fever (DHF), which can culminate in dengue shock syndrome (DSS) and death [3]. The vast majority of severe dengue cases occur in children and are currently a leading cause of hospital admission and death among children in Asia [4]. The transmission has been estimated to occur in up to 124 countries [5], with at least 50 million infected cases per year [6], including 250,000 cases of DHF/DSS [7]. A recent publication claims that this number may be as high as 390 million [8].

There are a number of factors associated with DHF [9], including host-related factors. Low platelet count is known to be a characteristic clinical manifestation in severe dengue, and a platelet count of less than 50,000/mm^3^ on days 5 to 7 after the onset of illness is associated with the development of complications, such as bleeding and shock [10, 11].

A recent genome-wide association study (GWAS) of hematological and biochemical traits identified six SNPs associated with low platelet count in healthy subjects [12]. These SNPs might also affect susceptibility to severe dengue. In the present study, to elucidate genetic factors in the host that influence the pathogenesis of dengue infection, the association of SNPs involved in low platelet count was examined in patients with dengue.

## Methods

### Patients

A total of 918 Thai patients with dengue who were treated at Ratchaburi Hospital and Lampang Hospital, Thailand, between 1999 and 2004 were investigated. All patients were ≤15 years old at diagnosis. They were confirmed as having DEN infection by dengue IgM/IgG capture enzyme-linked immunosorbent assay (ELISA), reverse transcriptase polymerase chain reaction (RT-PCR), and/or DEN isolation at the Arbovirus Laboratory, National Institute of Health, Department of Medical Sciences, Ministry of Public Health, Thailand. The patients were classified into two groups, DHF and DF, according to the WHO 1997 criteria [3]. In this study, DSS (i.e., DHF grades III and IV) is regarded as DHF. In brief, DF was defined as an acute illness with various nonspecific symptoms (e.g., headache, myalgia, arthralgia, rashes, and leucopenia). Patients with DHF had hemorrhagic manifestations, plasma leakage, and thrombocytopenia. Thrombocytopenia was diagnosed by platelet count of less than 100 000 per mm^3^ and a greater than 20 % increase in the packed cell volume from the baseline.

### Genotyping

Genomic DNA was extracted from peripheral blood leukocytes using the QIAamp Blood Kit (Qiagen, Hilden, Germany). Patients with dengue were genotyped for five SNPs, rs5745568 in *BAK1*, rs6141 in *THPO*, rs6065 in *GP1BA*, rs739496 in *SH2B3*, and rs385893 in *RCL1*, using TaqMan SNP genotyping assays (Applied Biosystems, Foster City, CA, USA). These SNPs were reported to be associated with low platelet count in a recent GWAS [12]. Although rs7775698 (*HBS1L*) also showed a significant association with low platelet count [12], it was not analyzed in the present study because the TaqMan probe was not available. In addition, rs4804803 in *CD209* was also genotyped to examine the possible interaction with rs5745568 in *BAK1*.

### Statistical analysis

The deviation from Hardy–Weinberg equilibrium was examined in both DHF and DF groups using a web tool [13]. To assess the association of each SNP with severe dengue, allele frequencies were compared using a Chi-square test between the two groups. The allelic odds ratio (OR) and the 95 % confidence interval (95 % CI) were calculated for the allele that was previously shown to be associated with low platelet count [12], which allowed us to examine the consistency in the direction of association, because low platelet count is likely to increase the risk for DHF in patients with dengue. Dominant and recessive models were examined for rs5745568 in *BAK1*. To control possible confounding factors, such as age, sex, hospital (i.e., geographic region), immune status (i.e., primary or secondary infection), and virus serotype, a logistic regression analysis adjusted for these independent variables was performed. In this analysis, the number of risk alleles (i.e., 0, 1, and 2) was used as an independent variable. The significance level was set at *P* < 0.05. SNPinfo [14] was used for *in silico* predictions of functional significance and for detection of SNPs in linkage disequilibrium (LD) with rs5745568 (*r*^2^ ≥ 0.8) in the HapMap-CHB and HapMap-JPT populations [15, 16].

## Results and discussion

A total of 918 confirmed patients with dengue, including 509 patients with DHF and 409 patients with DF, were investigated in this study (Table 1). They were genotyped for six SNPs. Of which, five SNP, rs5745568 in *BAK1*, rs6141 in *THPO*, rs6065 in *GP1BA*, rs739496 in *SH2B3*, and rs385893 in *RCL1*, were previously reported to be associated with low platelet count [12]. Although rs7775698 (*HBS1L*) also showed a significant association with low platelet count in a previous GWAS [12], it was not analyzed in the present study because rs7775698 could not be successfully genotyped. The genotype and allele frequencies of the SNPs in each dengue group are shown in Table 2. No SNP showed a significant deviation from the Hardy-Weinberg equilibrium in either dengue group. Among SNPs examined in this study, rs5745568 in *BKA1* was the only SNP that was significantly associated with a risk for DHF [*P* = 0.006 and crude OR (95 % CI) = 1.32 (1.09–1.60)]. A further analysis based on the genotype revealed that the GG genotype of rs5745568 significantly increased a risk for DHF compared to GT and TT [*P* = 0.003 and crude OR (95 % CI) = 1.50 (1.15–1.95)], while GG and GT did not significantly increase a risk compared to TT [*P* = 0.279 and crude OR (95 % CI) = 1.26 (0.83–1.93)], indicating that a recessive model best explains the association of G allele of rs5745568 with DHF. The association of rs5745568-G with DHF was also significant in a logistic regression analysis adjusted for age, sex, hospital, immune status, and virus serotype [*P* = 0.016 and adjusted OR (95 % CI) = 1.29 (1.05–1.58)]. The other SNPs showed no significant association in the logistic regression analyses (Table 2).

**Table 1:** Patient characteristics (total *n* = 918)

Characteristic	DHF *n* = 509	DF *n* = 409
Sex		
Male	276	213
Female	233	196
Hospital		
Lampang	134	172
Ratchaburi	375	237
Immune status		
Primary	65	94
Secondary	444	314
Serotype of virus		
D1	151	153
D2	197	109
D3	79	92
D4	81	54
Age^a^ (years)	10 (0–15)	9.0 (1–15)

^a^ Median (minimum–maximum)

**Table 2:** Association of SNPs with DHF

SNP ID (gene)	Genotype (Allele)	Patients with dengue		Chi-square test		Logistic regression analysis	
		DHF	DF	Crude OR (95 % CI)	*P*-value	Adjusted OR (95 % CI)	*P*-value
rs5745568 (*BAK1*)	TT	48	47				
	GT	191	187				
	GG	265	173				
	G-allele	721 (71.5 %)	533 (65.5 %)	1.32 (1.09–1.60)	0.006	1.29 (1.05–1.58)	0.016
rs6141 (*THPO*)	TT	82	70				
	CT	260	189				
	CC	164	148				
	C-allele	588 (58.1 %)	485 (59.6 %)	0.94 (0.78–1.14)	0.520	0.98 (0.80–1.19)	0.844
rs6065 (*GP1BA*)	TT	2	0				
	CT	37	42				
	CC	467	366				
	C-allele	971 (96.0 %)	774 (94.9 %)	1.29 (0.83–2.00)	0.263	1.35 (0.85–2.14)	0.203
rs739496 (*SH2B3*)	AA	18	12				
	AG	137	114				
	GG	350	281				
	A-allele	173 (17.1 %)	138 (17.0 %)	1.01 (0.79–1.29)	0.921	0.96 (0.74–1.24)	0.731
rs385893 (*RCL1*)	TT	49	39				
	CT	200	165				
	CC	258	205				
	T-allele	298 (29.4 %)	243 (29.7 %)	0.98 (0.80–1.21)	0.882	1.02 (0.83–1.25)	0.886
rs4804803 (*CD209*)	AA	411	323				
	GA	92	77				
	GG	4	8				
	G-allele	100 (9.8 %)	93 (11.4 %)	0.85 (0.63–1.15)	0.288	0.83 (0.61–1.13)	0.242

Frequencies are shown in parentheses (%). The odds ratio (OR) of rs5745568 in *BAK1*, rs6141 in *THPO*, rs6065 in *GP1BA*, rs739496 in *SH2B3*, and rs385893 in *RCL1* was calculated for the allele associated with low platelet count in a previous GWAS [12]. The OR of rs4804803 in *CD209* was calculated for the G allele. Genotype frequencies at SNPs examined did not deviate from those expected from the Hardy–Weinberg equilibrium in DHF and DF groups

Several studies have shown significant association of a *CD209* promoter SNP (rs4804803) with DF or DHF [17–19]. To examine if a *CD209* promoter SNP affects the association of *BAK1* SNP with DHF, we analyzed dengue patients for rs4804803. The rs4804803 SNP was not significantly associated with DHF in this study (Table 2). A logistic regression analysis adjusted for age, sex, hospital, immune status, virus serotype, and genotype of rs4804803 revealed that rs5745568 was significantly associated with DHF [*P* = 0.0167 and adjusted OR (95 % CI) = 1.29 (1.05–1.58)]. Thus, we conclude that the association of rs5745568 with DHF is independent of rs4804803.

Although the direct association of rs5745568-G with low platelet count in patients with dengue was not examined in this study, rs5745568-G has been reported to be associated with low platelet count in healthy subjects [12]. Together with our results, the low-level constitutive production of platelets caused by the G allele of rs5745568 may increase the risk of bleeding in dengue infection. To fully understand the biological significance of rs5745568 in the pathogenesis of dengue infection, the difference in the time course of the platelet count among dengue patients with different genotypes requires to be studied in future.

The rs5745568 SNP is located upstream of *BAK1*. To estimate the functional significance of rs5745568, an *in silico* analysis [14] was performed. The rs5745568 was predicted to be located in the putative binding sites of various transcription factors. Although the expression level of *BAK1* remains to be studied in patients with dengue, the *BAK1* expression level may vary among genotypes of rs5745568. At this locus, a total of eight SNPs were in LD (*r*^2^ ≥ 0.8) with rs5745568 in the HapMap-CHB or HapMap-JPT population (data not shown), which raises the possibility that the significant association of the G allele of rs5745568 with a risk for DHF may be explained by another causative variant in LD with rs5745568. Thus, it should be noted that rs5745568 may not be primarily associated with DHF.

BCL-2 homologous antagonist/killer (BAK), encoded by *BAK1*, is a member of the BCL-2 family of apoptosis regulators, including pro-apoptotic (BAX, BAD, BAK, and BOK) and anti-apoptotic (BCL-2, BCL-XL, and BCL-W) proteins. Bak is responsible for mediating platelet death in mice [20], which implies that the G allele of rs5745568 may be associated with a higher expression level of BAK in patients with dengue as well as in healthy individuals. The present results suggest that BAK plays a crucial role in the pathogenesis of DHF and is a potential target of therapy for DHF.

## Conclusions

The G allele of rs5745568 in *BAK1* was significantly associated with a risk for DHF. Although the association of the G allele of rs5745568 with low platelet count in patients with dengue remains to be confirmed in future studies, the present results suggest that the BAK protein, encoded by *BAK1*, plays a crucial role in the pathogenesis of DHF.
